# Using Mendelian Randomization to Study the Role of Iron in Health and Disease

**DOI:** 10.3390/ijms241713458

**Published:** 2023-08-30

**Authors:** Tara Zeitoun, Ahmed El-Sohemy

**Affiliations:** Department of Nutritional Sciences, Temerty Faculty of Medicine, University of Toronto, Medical Sciences Building, Room 5326A, 1 King’s College Circle, Toronto, ON M5S 1A8, Canada; tara.zeitoun@mail.utoronto.ca

**Keywords:** iron, Mendelian randomization, *HFE*, *TMPRSS6*, iron overload, cardiometabolic disorders, neurological disorders, inflammation, nutrigenetics, nutrigenomics

## Abstract

Iron has been shown to play a dual role in health and disease, with either a protective or harmful effect. Some of the contradictory findings from observational studies may be due to reverse causation, residual confounding, or small sample size. One approach that may overcome these limitations without the high cost of randomized control trials is the use of Mendelian randomization to examine the long-term role of iron in a variety of health outcomes. As there is emerging evidence employing Mendelian randomization as a method of assessing the role of micronutrients in health and disease, this narrative review will highlight recent Mendelian randomization findings examining the role of iron in cardiometabolic disorders, inflammation, neurological disorders, different cancers, and a number of other health-related outcomes.

## 1. Introduction

Iron is an essential trace element that is critical to the existence of almost all living organisms. Iron is required for the function of haemoglobin, erythrocytes that transport oxygen from the lungs to tissues, and myoglobin, which transports and stores oxygen in skeletal muscle tissue. This mineral is also required for physical growth, brain development, cellular function, and hormone production [1]. Iron’s capacity to transition between two thermodynamically stable oxidation states, Fe^3+^ or ferric form, and Fe^2+^ or ferrous form, makes it an ideal candidate for catalysis of biochemical processes, and a vast number of enzymes rely on iron for biological activity [2]. This metal is also able to catalyze reactions leading to the production of toxic oxygen radicals when present in excess. Recent developments in the field of iron metabolism have revealed multiple complex pathways that are crucial for the upkeep of iron homeostasis. However, iron has a dual nature, in which deficiency may lead to certain symptoms and potentially to iron deficiency anaemia, while excess iron may cause symptoms of toxicity [3]. Though iron deficiency is the most prevalent micronutrient deficiency worldwide [4], assessing the duality of iron levels is necessary to prevent both the unwanted adverse effects of either deficiency or toxicity.

Nutritional epidemiology seeks to uncover dietary and lifestyle factors that contribute to human health and disease [5]. Most evidence linking nutrition to risk of disease is based on data from observational studies, which can be distorted by reverse causation and unmeasured or residual confounding. Mendelian randomization (MR) is an approach used in genetic epidemiology that combines genetic and traditional epidemiological research to infer causation of environmental exposures [6]. This technique uses genetic variants as proxy measures for exposures of interests. The use of genetic determinants of iron status to study the long-term effects of iron, can help clarify the role of iron in the etiology of various diseases, and may lead to novel screening measures that can be used to prevent iron deficiency/toxicity. This review summarizes recent advances in the application of MR to assess the role of iron in multiple health outcomes and disease states.

## 2. Iron Requirements

The recommended dietary allowance (RDA) of iron for adults aged 19–50 years is 8 mg/day for men, 18 mg/day for women, 27 mg/day during pregnancy, and 9 mg/day during lactation [7]. The RDA drops for women over 51 years to 8 mg/day due to the assumption of the cessation of menstruation from menopause. The tolerable upper intake level, defined as the maximum daily intake that is unlikely to cause harmful effects on health, is 45 mg/day for all males and females over 14 years of age [7]. Dietary iron is available in haem and non-haem forms, and each has a separate and distinct mode of uptake by enterocytes. Haem iron is highly bioavailable, comes from animal sources such as meat, fish and poultry, and is less influenced by dietary constituents. Non-haem iron is less bioavailable and comes from plant sources such as legumes and leafy greens and is also the form found in most supplements. The bioavailability of iron is around 14–18% in mixed diets that include animal products and vitamin C rich foods and 5–12% in vegetarian diets [1]. The absorption of iron is related to iron stored in ferritin, such that absorption is inversely proportional to serum ferritin concentrations [8].

## 3. The Duality of Iron Levels

Excess and deficient states of iron are harmful to human health and can lead to a plethora of health concerns. Disorders in the hepcidin and ferroportin regulatory systems cause an imbalance in iron metabolism, leading to either iron deficiency or overload. Iron status differs between the sexes, with iron deficiency seen more frequently in females than in males due to a variety of factors, including amount of iron consumed, use of hormonal contraceptives, hormone replacement therapy, and menstruation [9,10]. Iron overload is more frequently observed in middle-aged males with hereditary haemochromatosis compared to females due to the amount of iron consumed and accumulated [10].

Iron toxicity may be acute, resulting from a single large ingestion, or chronic, due to the long-term accumulation of iron in the body, which is referred to as iron overload. Some common symptoms of iron toxicity include nausea and chronic fatigue and, if progressed, can lead to liver and heart damage, diabetes, certain kinds of cancer, and chronic cardiometabolic conditions [2]. Haemochromatosis is an iron disorder characterized by increased iron absorption and excessive iron build-up in the body. There are two main manifestations of haemochromatosis: primary haemochromatosis, which is hereditary, and secondary haemochromatosis, which is a result of ineffective erythropoiesis or chronic blood transfusions [11]. Primary haemochromatosis, also known as hereditary haemochromatosis (HH), is typically caused by a mutation in the haemostatic iron regulator (HFE) gene, which results in an excessive build-up of iron in the body and is the most common form of haemochromatosis [12]. Secondary haemochromatosis results from iatrogenic iron administration, haematologic conditions, chronic blood transfusions and liver disease [13]. The clinical symptoms of hereditary haemochromatosis appear in post-menopausal females due to the cessation of blood loss experienced during menstruation, childbirth, and lactation, which compensate for the increased absorption [14]. Clinical symptoms of haemochromatosis occur earlier in males, most commonly in the 40–50 age range [15].

In females of reproductive age, iron deficiency and iron deficiency anaemia are most likely caused by menstrual blood losses and pregnancy blood losses [16,17]. The clinical presentations of iron deficiency anaemia include weakness, irritability, fatigue, hair loss, poor concentration, and pica [18]. Although the etiology of iron deficiency anaemia is mainly related to low iron intake, genetic variations that impair absorption may also play a role [19]. Pregnant women, infants and young children, women with heavy menstrual bleeding, frequent blood donors, and those with cancer, heart failure or gastrointestinal disorders are among those at high risk of iron deficiency.

## 4. Iron Overload Genes

**Figure 1:**
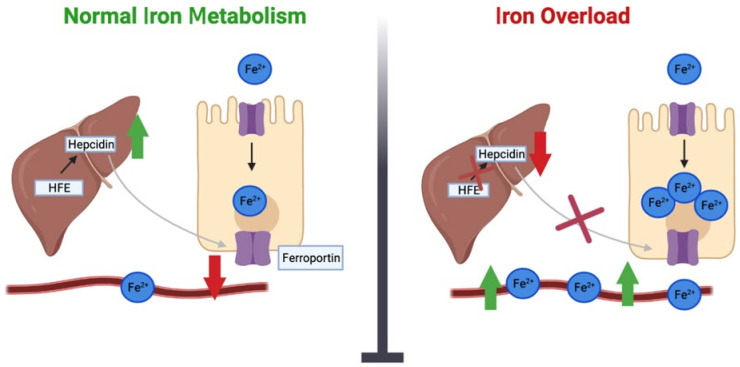
Under conditions of normal iron metabolism, iron is absorbed by enterocytes and exits enterocytes via ferroportin, which is regulated by the hormone hepcidin. The liver secretes hepcidin, whose main role is to keep iron levels balanced. Hepcidin is regulated by HFE. With iron overload, there is decreased expression of the *HFE* gene. As a result, HFE is unable to regulate hepcidin, which leads to a decrease in hepcidin levels. This in turn activates the ferroportin channel, allowing an efflux of iron into the blood and tissues and leading to a state of iron overload.

Iron homeostasis is, in part, affected by variations in the homeostatic iron regulator (*HFE)* gene [20]. As depicted in Figure 1, *HFE* detects the amount of iron in the body by triggering the production of hepcidin to maintain levels of iron production [21]. Hepcidin is a key regulator of cellular iron balance and manages the control of the iron export protein ferroportin. Low hepcidin contributes to excessive expression of ferroportin at the cell surface, which results in increased plasma iron and increased transferrin saturation [22]. A decrease in hepcidin further increases the absorption of iron from the intestine and increases the storage of iron in the liver and other tissues. Variations in the *HFE* gene have been associated with a reduction in hepcidin release, which can lead to iron overload and the inherited disorder hereditary haemochromatosis (HH) [12,23]. HH is recognized as one of the most common autosomal recessive genetic disorders in the northern European population [20].

There are many single-nucleotide polymorphisms (SNPs) within *HFE*, but this review will focus on the most common SNPs that have been associated with the development of hereditary haemochromatosis: rs1800562, rs1799945, and rs1800730 [24]. The most common SNP, rs1800562, consists of a guanine-to-adenine substitution (G→A) at nucleotide 845, resulting in a replacement of cytosine by tyrosine at amino acid 282 of the HFE protein [25]. This C282Y polymorphism prevents the HFE protein from reaching the cell surface and further prevents interaction with hepcidin and transferrin receptors. Carrying two copies of the A allele in rs1800562 is associated with a reduction in hepcidin release, leading to an excessive increase in blood levels of iron and transferrin. The second most common *HFE* polymorphism, the rs1799945 SNP, is characterized by a cytosine-to-guanine transversion (C→G) at nucleotide 187, changing the amino acid histidine into aspartic acid at position 63 of the protein, and is also referred to as H63D [26,27]. The H63D polymorphism is not as penetrant as the C282Y polymorphism, but individuals who are homozygous for the G allele of H63D are more likely to be affected by a mild form of haemochromatosis [28]. A third common SNP in the *HFE* gene responsible for HH is rs1800730, which is characterized by an adenine-to-tyrosine (A→T) substitution at nucleotide 193, where serine is replaced by cytosine at position 65 of the protein and is known as the S65C polymorphism [29,30]. Homozygotes for the T allele of rs1800730 are affected by an increased risk of a mild form of haemochromatosis when in combination with the heterozygosity C282Y polymorphism.

Iron overload has been reported in those who have digenic inheritance of one or more *HFE* mutations [21]. The C282Y polymorphism of the *HFE* gene is responsible, alone or in combination with H63D or S65C, for up to 85–90% of hereditary forms of iron overload among northern European populations [12]. The remaining 10–15% of individuals with hereditary iron overload most likely have other mutations in the *HFE* gene or other genes involved in iron homeostasis [29]. In northern Europeans, the most common genotype is C282Y homozygotes, carriers of which are predisposed to HH alongside unmonitored diet and to some extent alcohol abuse [31]. Around 1 in 10 Caucasians carry the risk variant of the C282Y polymorphism, but only 4 in 1000 appear to be homozygous and present clinical signs of haemochromatosis [32]. The H63D variant is more prevalent in the general population and is less subject to specific geographic locations but is typically associated with more severe iron loading when present in combination with the C282Y risk variant [31].

## 5. Low Iron Genes

Iron deficiency can arise over a long time where there is a chronic condition that reduces iron absorption (such as chronic inflammation) or chronically low intake of iron-rich foods. Evidence suggests that some individuals with iron deficiency anaemia do not respond to oral iron therapy and only moderately respond to parenteral iron administration, suggesting that iron deficiency may also be genetically resolute [19]. This phenotypic expression of iron deficiency is also clinically known as iron-refractory iron deficiency anaemia. The more commonly assessed genes in this form of iron deficiency are *TMPRSS6* and *TFR2. TMPRSS6* encodes a type II transmembrane serine protease or matriptase-2, and it controls the expression of hepcidin to maintain levels of iron absorption in the gut [33,34]. When the expression of *TMPRSS6* is reduced, there are high levels of haemojuvelin, which leads to overexpression of hepcidin in the liver [35]. An elevation of hepcidin production prevents the release of iron from cells, leading to low iron in the blood [36]. The *TFR2* gene, coding for transferrin receptor 2 protein, helps iron enter the cell [36]. The *TF* gene, coding for the protein transferrin, is responsible for transporting iron in the body [37].

The minor allele of the rs4820268 SNP in *TMPRSS6* consists of a cytosine replaced by a thymine (C→T) at nucleotide position 1563, which leads to a synonymous change of aspartic acid at position 521 of the protein (D521D). The rs4820268 polymorphism has been found to alter the hepcidin iron feedback loop, resulting in a risk of low transferrin saturation, which can lead to risk of low iron stores in individuals homozygous for the G allele [26]. The rs855791 SNP consists of a cytosine replaced by a thymine (C→T) at nucleotide 2207, which results in a missense shift of valine to alanine at position 736 (V736A) near the catalytic and binding site of the matriptase-2 protein [38]. The rs855791 polymorphism is also used to assess iron parameters in the general population [33,34]. Individuals homozygous for the C allele of the rs855791 polymorphism appear to be protected against iron deficiency anaemia compared to those homozygous for the T allele, who had 0.2 g/dL lower haemoglobin on average [39,40,41]. For the *TFR2* gene, those homozygous for the A allele at rs7385804 were found to have a greater risk of low ferritin and elevated transferrin levels [26]. For the rs3811647 within *TF*, those with the CC genotype have an increased risk of low serum ferritin levels [26].

## 6. Mendelian Randomization

MR is an analytical method from genetic epidemiology that uses genetic variants, which are fixed and randomly assigned at conception, as proxy variables for modifiable risk factors that affect health and disease [42,43]. Its use is increasing due to the proliferation of genome-wide association studies (GWAS) and because it has several strengths that can overcome some of the major limitations of observational studies in nutritional epidemiology. MR is a method that uses genetic markers that are robustly associated with exposures to examine their effects on a health or disease outcome [6,44]. Due to the nature of MR, the association between the genetic variant and health outcome is not likely to be confounded by behavioral or environmental exposures, or reverse causations [45].

Many studies have now examined the relationship between iron and chronic diseases using MR. Though the gold standard for inferring causality in epidemiology is randomized controlled trials (RCTs), such studies give little insight into the long-term exposures due to their short follow-up times [5]. The use of genetics to infer non-genetic risk factors (such as micronutrient exposure) is a feasible method, since these genetic associations overcome many of the limitations of observational studies.

There are two main forms of MR methods: one-sample and two-sample MR. One-sample MR is characterized by the use of variables and measures of genotypes and outcomes from the same sample of individuals [46]. One-sample MR is typically seen as a simpler way of utilizing MR due to the nature of using a single dataset which may be more straightforward to analyze. Some of the limitations of one-sample MR are weak instrument bias, measurement error, and population-specific effects. Two-sample MR utilizes two separate study populations or datasets to examine the instrument–risk factor and instrument–outcome associations [46]. This type of MR analysis is more advantageous than one-sample as it can utilize summary results from GWAS, which includes more data with more precision and higher statistical power. Moreover, a two-sample MR analysis does not require the risk factor and outcome to be measured in a single study, which may be expensive or difficult to measure depending on the study question.

There are many limitations to the application of MR methodology. The most obvious limitation is that if an exposure lacks a functional genetic variant as a proxy measure, MR cannot be carried out. Population stratification, or the systematic difference in allele frequencies between subpopulations is another limitation of MR that may lead to false positive associations [42,47]. This may be avoided by restricting analyses to ethnically homogenous groups. At times, it may also be possible for the genetic variant of interest to be in linkage disequilibrium with another polymorphic locus [48]. This is another limitation of MR, where non-random associations between the locus under study and other genetic variants at different loci on the same chromosome exist [6]. Linkage disequilibrium can be tested using multiple established statistical methods. To mitigate some of these limitations, sensitivity analysis such as MR–Egger regressions can be carried out. MR–Egger regression is a sensitivity analysis method which accounts for a way to provide causal estimates even when faced with pleiotropy [6]. Although there are statistical methods aimed at overcoming MR’s common limitations, residual biases could persist.

**Figure 2:**
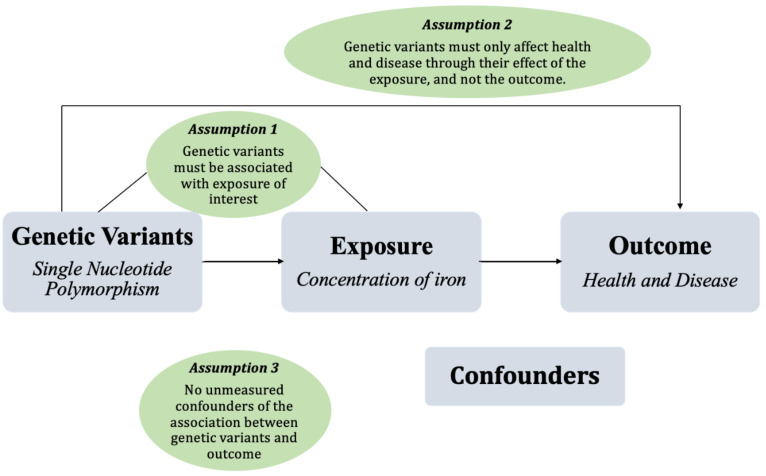
Depiction of mendelian randomization analysis when assessing the role of iron in health and disease.

The primary assumptions when utilizing MR as an investigative method are depicted in Figure 2. The three assumptions of utilizing MR for a study to be valid are as follows: (1) the genetic variants must be associated with the exposure of interest; (2) the genetic variants must affect the outcome solely through their effect on the exposure of interest, and not the outcome as well, thus ruling out horizontal pleiotropy; and (3) there must be no unmeasured confounders of the associations between genetic variants and the outcome. Generally, these three key assumptions must be met for an analysis to be valid, and if one of these is violated, any inference regarding the causal effect would be biased [45,46].

## 7. Mendelian Randomization, Iron, and Health

MR studies employing data from large cohorts support a causative role for iron in many diseases. This review will summarize the studies using MR to examine the role of iron in cardiometabolic disorders, oncology, neurology, inflammation, and additional health outcomes; a tabular summary of these studies can be found in the Table S1 in Supplementary Material. The role of iron in health can first be assessed by examining its role in all-cause mortality and life expectancy, both of which have been examined using MR. Using data from the United Kingdom (UK) Biobank and LifeGen consortium, there is evidence that higher genetically predicted iron status is causally related to reduced life expectancy determined by using parental lifespan as a life expectancy marker [49]. Further research found that increased serum iron and transferrin saturation may contribute to all-cause mortality [50]. Despite these deleterious effects, the role of iron in health is contradictory, and higher iron is causally protective for some diseases, yet harmful for others.

## 8. Cardiometabolic Disorders

Cardiometabolic disorders (CMD), which include, but are not limited to, cardiovascular disease (CVD), hypertension, dyslipidemia, and obesity, are among some of the main causes of morbidity and mortality [51]. There are, however, sex differences in the timeline for CMDs. Females have an advantage in their younger years by having lower rates of CMD compared to males. After menopause, CMD rates in women start to rise, and one of the hypotheses for this age-related occurrence is iron metabolism and naturally occurring iron levels in women due to the cessation of menstruation [52]. However, low iron status is associated with iron deficiency anaemia, which is a risk factor for coronary heart disease [53]. Furthermore, the effects of iron on CMDs differ based on the type of disorder; thus, it can be difficult to disentangle the relationship between iron and CMDs.

### 8.1. Type 2 Diabetes, Obesity and Hypertension

One of the risk factors for CMDs, central obesity, has been observed to be causally associated with increased liver iron content due to the H63D and C282Y variants in the *HFE* gene and the rs855791 SNP from the *TMPRSS6* gene [54]. Findings from [54], and others also demonstrate an association between the risk variant of *HFE* C282Y and higher risk of type 2 diabetes, indicating that higher iron levels may not be protective against type 2 diabetes [54,55]. The authors also observed an association between the risk variant of *HFE* H63D and hypertension [54]. As hypertension is a risk factor for atrial fibrillation, data from GWAS consortia studies have also indicated that higher genetically predicted iron levels are associated with an increased risk of atrial fibrillation [56].

### 8.2. Cholesterol

Not all genetically predicted levels of high iron result in deleterious effects on cardiovascular health, as protective effects of the *HFE* genotypes and total cholesterol and low-density lipoprotein (LDL) cholesterol have previously been identified [54]. Furthermore, the *TMPRSS6* SNP rs855791, associated with lower iron stores, is associated with a lower risk of ischaemic heart disease, angina pectoris, and hyperlipidemia [54]. Some of these findings were further replicated in another study, where genetically predicted iron status using the same three variants was also associated with lower risk of hyperlipidemia and hypercholesterolemia and lower total- and LDL-cholesterol [57].

### 8.3. Heart Failure and Stroke

Heart failure risk, a clinical syndrome of cardiac dysfunction, was not previously associated with genetically predicted iron status [58]. Despite these findings, another MR study also recently reported that the same genotypes, H63D, C282Y and the rs855791 SNP from *TMPRSS6*, may have a protective effect against coronary artery disease [59], suggesting that higher iron levels may be protective. Data from the Genetics of Iron consortium demonstrated that genetically determined iron status predicted detrimental effects of iron on stroke risk, where a positive association with the iron overload genes was observed, and an inverse association with the low iron status genes was observed [60].

### 8.4. Vascular Disease

Although iron status has been consistently observed to be protective against coronary artery disease, in the wider context of vascular diseases, this association remains inconsistent. Research assessing vascular diseases from the UK Biobank cohort also suggested protective effects of the increased iron status genes against the risk of coronary atherosclerosis in males, but a detrimental effect on varicose veins in both sexes [61]. The latter results were also observed in the UK Biobank and FinnGen consortia, where increased iron levels were also associated with an increased risk of varicose veins [62]. Findings from the Genetics of Iron consortium also indicate that genetically determined higher iron levels, resulting from *HFE* (C282Y and H63D) and *TMPRSS6,* are protective against some forms of atherosclerotic diseases, but increase the risk of thrombosis related to stasis of blood, further demonstrating a conflicting relationship between iron status and vascular pathology [63]. According to these controversial findings, further studies are required to verify the role of iron on vascular pathology. Though the reason for these contradictory findings remains unclear, it is possible that iron may have a divergent role in CMDs, serving as a protective factor in some forms and a harmful one in others.

## 9. Neurological Disorders

Iron is involved in many vital processes in the central nervous system, including but not limited to oxygen transport, myelin production, metabolism of neurotransmitters, and oxidative phosphorylation. Moreover, as evidenced by a GWAS of brain MRI scans, *HFE* C282Y has been found to be associated with iron accumulation in parts of the brain [64]. Iron deficiency causes neuropsychologic impairment and behavioral dysfunction and also contributes to long-term changes such as dopamine metabolism, myelination, and hippocampal structure and function [65].

Previous research from the UK Biobank cohort found the iron overload *HFE* C282Y genotype to be associated with multiple sclerosis [54]. Findings using data from the UK Biobank and the FinnGen consortium indicate an association between high iron status and an increased risk of epilepsy [66]. Iron’s relationship with neurological conditions is not consistently deleterious. Distinctively, 2-sample Mendelian randomization research on iron and Parkinson’s disease (PD) shows a protective effect from iron on PD, with a 3% relative reduction in PD risk [67]. Research in women’s health has assessed the role of iron in the prevalence of psychological premenstrual symptoms. MR analysis conducted using female participants from the Toronto Nutrigenomics and Health Study found a protective role played by iron on premenstrual confusion and headaches, whereby women with a genetic predisposition to iron overload had a lower risk of experiencing those specific premenstrual symptoms [68]. Compared to studies using data from consortia, this study had a much smaller sample size and also did not test for pleiotropy [68].

## 10. Inflammation and Arthritis

Data from the UK Biobank cohort found the *HFE* C282Y genotype to be associated with osteoarthritis [54]. Increased plasma ferritin concentration, a marker of increased iron concentration, has been shown to be associated with low-grade inflammation, according to three independent Danish population-based studies [69]. This association may indicate a causal link between elevated ferritin and the *HFE* genotype, in addition to low-grade inflammation, CRP, and complement-3; however, this study may be limited by pleiotropy, as the *HFE* gene may have immunological effects. Genetic predisposition to high iron status was also causally associated with lower odds of rheumatoid arthritis in the UK Biobank [70]. Transferrin saturation (homozygotes for C282Y) is associated with increased knee osteoarthritis (OA) [71]. A study on serum nutritional factors and OA revealed that ferritin has no genetic association with knee OA and hip OA [72], which is consistent with findings from a previous study [71]. They did, however, find that iron levels were positively associated with the risk of OA in women, indicating a sex-specific difference [72]. Furthermore, back pain has also been seen to be associated with genetically instrumented high serum iron, ferritin, and transferrin saturation [73].

## 11. Oncology

Population-based studies that have examined the associations between serum iron and cancer outcomes are limited and have reported conflicting findings depending on the type of cancer. Higher iron seems to play a role in carcinogenesis, which may be due to the oxidative stress and catalytic activity of iron via the formation of hydroxyl radicals [74]. Iron also has the ability to suppress host defenses, which may promote cancer cell proliferation. Despite this, the research yields discordant findings between iron and cancer. Results from a previous meta-analysis shows an association between *HFE* C282Y and a higher risk of breast cancer, colorectal cancer, hepatocellular carcinoma, and total cancer in over 87,000 participants [75]. Such findings suggest a relationship between higher liver iron stores and cancer risk.

Further research from the UK Biobank demonstrates that genetically predicted iron levels were positively and significantly associated with an increased risk of different dimensions of liver injury, a precursor to liver cancer [76]. Findings from that study also demonstrate a sex-dependent causal association, in which the effect size for liver disease was larger in men. Despite these associations, Tian et al. investigated the relationship between primary liver cancer and *HFE/TMPRSS6* and found no association between the genetic markers for iron metabolism and liver cancer [77]. Conversely, in a 2-sample MR analysis, Yuan et al. reported that genetically predicted iron status was positively associated with liver cancer [78]. The differing conclusions may be due to the different numbers of SNPs used to serve as genetic instruments; Yuan et al.’s study included 3 SNPs, and the study by Tian et al. included many more, thus reaching GWAS significance [77].

Genetic predictors of iron status have also been associated with other types of cancers. Variations in the *HFE* and *TMPRSS6* genotype associated with higher iron stores were inversely associated with brain cancer but not with overall cancer [78]. Research using data from the Genetics of Iron consortium and International Lung Cancer consortium found no associations between genetically predicted iron status using *HFE, TMPRSS6* genes and lung cancer [79]. Serum transferrin levels were positively associated with breast cancer risk, but other measures of iron status were not related using 2- sample MR [80]. Moreover, no association between genetically predicted iron status and epithelial ovarian cancer risk found using data from the Ovarian Cancer Association GWAS [81]. Iron associated genetics were associated with reduced prostate cancer risk [82]. 

## 12. Other Conditions

Some health outcomes have not been investigated as thoroughly as the previous sections of this review. The role of iron in kidney function and disease was recently investigated in an MR study, using the UK Biobank and Genetics of Iron consortium data [83]. The authors found that genetic markers of iron status (H63D, C282Y from *HFE*, and rs855791 of *TMPRSS6*) had protective effects on kidney function, with a 1.3% increase in estimated glomerular filtration rate per standard deviation increase in iron [83]. MR analyses have also been conducted assessing the role of iron in sarcopenia, using *HFE* C282Y and H63D genotypes and *TMPRSS6* rs855791. Using participants from the UK Biobank study, iron was shown to be causally related to sarcopenia, in which each standard deviation of iron corresponded with a 53% increased risk of sarcopenia [84]. Though iron has been observed to play a role in the occurrence of skeletal muscle disorders, such as sarcopenia, elevated biomarkers of iron status in the *HFE* gene are more common in elite-level athletes compared to the general population, suggesting an advantageous effect on performance [85]. Indeed, in MR analysis, *HFE* risk genotypes were found to be associated with enhanced endurance performance and increased VO2 peak in male athletes [86]. Such findings give insight into the dual nature of iron, which in excess has harmful effects on muscle tissue, while the high end of a normal range may be beneficial.

## 13. Conclusions and Future Directions

This review describes a number of studies using MR to examine the role of iron in different conditions. There is a growing body of evidence around iron and health employing MR; however, additional studies are needed to better understand the divergent effects of iron on health and disease. One of the limitations of genetic markers of iron, such as the *HFE* gene for example, is that the risk variant is found primarily in those of northern European ancestry and may not apply to other ethnic groups, who may be at lower risk for high iron levels. As iron status is seen to differ between males and females, further research employing MR should continue to stratify by sex to better understand the different ways iron may affect health between the sexes.

## Supplementary Materials

The supporting information can be downloaded at: https://www.mdpi.com/article/10.3390/ijms241713458/s1.

## Abbreviations

MR: Mendelian randomization; RDA, recommended daily allowance; HFE, homeostatic iron regulator; HH, hereditary haemochromatosis; SNP, single nucleotide polymorphism; GWAS, genome wide association study; UK, United Kingdom; CMD, cardiometabolic disorders; CVD, cardiovascular disease; LDL, low-density lipoprotein; OA, osteoarthritis; PD, Parkinson’s disease.
