# Supplementary material for: Using Mendelian Randomization to Study the Role of Iron in Health and Disease
Source: Int J Mol Sci. 2023 Aug 30;24(17):13458. doi: 10.3390/ijms241713458 (PMC10487635; doi:10.3390/ijms241713458)
Supplement: Supplementary file 1 [file ijms-24-13458-s001.zip › ijms-2487409-supplementary.pdf]

**Table S1.** Summary of review findings.				
**Author**	**Sample**	**Exposure**	**Outcome**	**Findings**
Wilman, R., et al. 2019	*n* = 8,239 individuals from the UK Biobank.	*HFE* (rs1800562 and rs198851) *TMPRSS6* (rs855791)	25 metabolic traits	*HFE* C282Y was associated with higher liver fibrosis/cirrhosis, higher risk of type 2 diabetes, hypertension, alcohol-related liver disease, arthrosis, chronic and degenerative neurological conditions including multiple sclerosis, and but lower total cholesterol, lower LDL and lower BMI (FDR < 5%).<br><br>*HFE* H63D was associated with higher risk of hypertension, ankylosing spondylitis and bladder malignancy but lower risk of malabsorption or coeliac disease and lower cognitive ability (FDR <5%).<br><br>*TMPRSS6* was associated with lower risk of ischaemic heart disease, angina pectoris, and lipidaemias (FDR <5%).
Wang, X., et al 2021	*n* = 48,972 individuals from the Genetics of Iron Status consortium.<br><br>*n* = 74,125 individuals with diabetes from the DIAGRAM Study.	*HFE* (rs1800562 and rs198851) *TMPRSS6* (rs855791)	Type 2 Diabetes	Genetically instrumented serum iron (OR: 1.07; 95% CI, 1.02-1.12), ferritin (OR: 1.19; 95% CI, 1.08-1.32), and transferrin saturation (OR: 1.06; 95% CI, 1.02-1.09) were positively associated with T2D. Genetically instrumented transferrin, a marker of reduced iron status, was inversely associated with T2D (OR: 0.91; 95% CI, 0.87-0.96).
Wang, T., et al. 2022	*n* = 48, 972 individuals from the Genetics of Iron Status consortium<br><br>*n* = 588,190 individuals from the AFGen Consortium Study	*HFE* (rs1800562 and rs198851) *TMPRSS6* (rs855791)	Atrial Fibrillation	Genetically instrumented serum iron (OR: 1.09; 95% CI, 1.02–1.16), ferritin (OR: 1.16; 95% CI, 1.02–1.33), and transferrin saturation (OR: 1.05; 95% CI, 1.01–1.11) were positively associated with atrial fibrillation. Genetically instrumented transferrin levels (OR: 0.90; 95% CI, 0.86–0.97) were inversely correlated with atrial fibrillation.

Zhou, J., et al. 2020	*n* = 48, 972 individuals from the Genetics of Iron Status consortium<br><br>*n* = 310,999 from UK Biobank	*HFE* (rs1800562 and rs198851) *TMPRSS6* (rs855791)	Lipid Metabolism Disease Outcomes: hyperlipidemia, hypercholesterole mia, varicose veins.	Genetically predicted high iron status biomarkers were associated with lower hyperlipidemia (OR: 0.90; 95% CI, 0.85–0.96) and hypercholesterolemia (OR: 0.90; 95% CI, 0.84–0.95), and an enhanced risk of varicose veins (OR: 1.28; 95% CI, 1.15–1.42).
Wang, X., et al. 2022	*n* = 48, 972 individuals from the Genetics of Iron Status consortium<br><br>*n* = 361,194 individuals from the UK Biobank	*HFE* (rs1800562 and rs198851) *TMPRSS6* (rs855791)	Heart Failure	Genetically predicted iron status biomarkers<br>ferritin (OR: 0.99; 95% CI, 0.99-1.00),<br>iron (OR: 0.99; 95% CI, 0.99-1.00),<br>transferrin (OR: 1.00; 95% CI, 0.99-1.00) and<br>transferrin saturation (OR: 0.99; 95% CI, 0.99-1.00) were not causally associated with heart failure risk.
Gill, D., et al. 2017	*n* = 48, 972 individuals from the Genetics of Iron Status consortium<br><br>*n* = 84,509 individuals from the CARDIoGRAMplus C4D Consortium	*HFE* (rs1800562 and rs198851) *TMPRSS6* (rs855791)	Coronary Artery Disease	Genetically determined higher iron status biomarkers iron (OR: 0.94; 95% CI, 0.88-1.00), transferrin saturation (OR: 0.95; 95% CI, 0.91–0.99) log-transformed ferritin (OR: 0.85; 95%CI, 0.73–0.98) and transferrin (OR: 1.08; 95% CI, 1.01–1.16) were protective for coronary artery disease risk.
Gill, D., et al. 2018	*n* = 48, 972 individuals from the Genetics of Iron Status consortium<br><br>*n* = 446,696 individuals from the MEGASTROKE Consortium	*HFE* (rs1800562 and rs198851) *TMPRSS6* (rs855791)	Stroke	Genetically determined higher iron status biomarkers iron (OR: 1.07; 95% CI, 1.01-1.14), transferrin saturation (OR: 1.06; 95% CI, 1.01-1.11) log-transformed ferritin (OR 1.18; 95% CI, 1.02-1.36) were associated with increased risk of stroke. Higher transferrin (indicative of lower iron levels), was associated with decreased stroke risk (OR: 0.92; 95% CI, 0.86-0.99).
Yang, F., et al. 2020	*n* = 361,194 individuals from the UK Biobank<br><br>*n* = 48, 972 individuals from the	*HFE* (rs1800562 and rs198851) *TMPRSS6* (rs855791)	Vascular diseases	There was a detrimental effect of serum iron (OR: 1.005; 95% CI, 1.003-1.008), ferritin (OR: 1.012; 95% CI, 1.006-1.017), and transferrin saturation (OR: 1.004; 95% CI, 1.002, 1.006) on varicose veins of lower extremities.

	Genetics of Iron Status consortium			High serum levels of transferrin (indicated as the lower systemic iron) were associated with a decreased risk of varicose veins of lower extremities (OR: 0.995; 95% CI, 0.992-0.995).<br><br>Higher serum levels of iron (OR: 0.995; 95% CI, 0.992, 0.998), ferritin (OR: 0.992; 95% CI, 0.985, 0.998) and transferrin saturation (OR: 0.997; 95% CI, 0.995, 0.999) were associated with a decreased risk of coronary atherosclerosis.
Yuan, S., et al. 2021	*n* = 361,194 individuals from the UK Biobank<br><br>*n* = 167,879 individuals from the FinnGen Consortium	*HFE* (rs1800562 and rs198851) *TMPRSS6* (rs855791) *TF* (rs8177241)	Varicose veins	Genetically predicted iron status was associated with an increased risk in varicose veins (OR: 1.24; 95% CI, 1.16–1.33).
Gill, D., et al. 2019	*n* = 48, 972 individuals from the Genetics of Iron Status consortium<br><br>*n* = 60,139 individuals from the International Network on Venous Thrombosis Consortium	*HFE* (rs1800562 and rs198851) *TMPRSS6* (rs855791)	Venous thromboembolism and carotid atherosclerotic disease	Genetically determined high iron status was positively associated with risk of venous thromboembolism. OR per SD increase in biomarker levels were 1.37 (95% CI 1.14-1.66) for serum iron, 1.25 (1.09-1.43) for transferrin saturation, 1.92 (1.28-2.88) for ferritin, and 0.76 (0.63-0.92) for serum transferrin.<br><br>Higher iron status by serum iron (OR: 0.85; 95% CI, 0.73-0.99) and transferrin saturation (OR: 0.89; 95% CI, 0.80,1.00) was associated with lower risk of carotid plaque.
Yuan, S., et al. 2021	*n* = 361,194 individuals from the UK Biobank<br><br>*n* = 149,368 individuals from the FinGenn consortium	*HFE* (rs1800562 and rs198851) *TMPRSS6* (rs855791) +*ADD TF*	Epilepsy	Genetically determined iron status was positively associated with epilepsy. The odds ratios of epilepsy were 1.44 (95% CI, 1.13, 1.85) for one standard deviation increase in serum ferritin, 1.12 (95% CI, 1.04, 1.21) for one standard deviation increase in transferrin saturation.

Pichler, I., et al. 2013	*n* = 21,567 individuals from the Genetics of Iron Consortium.<br><br>*n* = 109,701 individuals from the PDGene Database, PD GWAS Consortium, 23andMe, and IPDGC.	*HFE* (rs1800562 and rs198851)<br>*TMPRSS6* (rs855791)	Parkinson's Disease	Genetically determined (combined) iron status was associated with a decreased risk in Parkinson's disease (OR: 0.997; 95 %CI, 0.994,0.999).
Zeitoun, T., et al. 2021	*n* = 254, Toronto and Nutrigenomics Health Study	*HFE* (rs1800562 and rs198851)	Premenstrual symptoms	Compared with participants with typical risk of iron overload, those with an elevated risk of iron overload were less likely to experience premenstrual symptoms of confusion (OR: 0.13; 95% CI: 0.02, 1.00), headaches (OR: 0.28; 95% CI: 0.08, 0.98), and nausea (OR: 0.13; 95% CI: 0.02, 0.99).
Moen, I., et al. 2018	*n* = 62,537 individuals of Danish descent from the Copenhagen City Heart Study examination the Copenhagen General Population and the Danish General Suburban Population Study	*HFE* (rs1800562 and rs198851)	Low-grade inflammation (CRP and complement 3)	Genetically determined plasma ferritin was positively associated with low-grade inflammation. The OR and (95% CI) for CRP and complement 3 were specifically. 1.03 (1.01–1.06) and 1.06 (1.03–1.12), respectively.
Yuan, S., et al, 2020	*n* = 48,978 individuals from the Genetics of Iron Consortium.<br><br>*n* = 140,000 individuals from the Global Urate Genetics Consortium	*HFE* (rs1800562 and rs198851)<br>*TMPRSS6* (rs855791)<br>*TFR & TF*	Gout, Arthritis, and inflammatory disease	Genetic predisposition to high iron status was causally associated with higher odds of gout, lower odds of rheumatoid arthritis, but not inflammatory bowel disease. The ORs of gout were 1.35 (95% CI, 1.00- 1.81), 2.07 (95% CI, 1.23- 3.50), 1.27 (95% CI, 1.07- 1.50) and 0.69 (95% CI, 0.54- 0.90) per one standard deviation increment of serum iron, ferritin,

				transferrin saturation, and transferrin levels, respectively. For rheumatoid arthritis, the corresponding ORs were 0.79 (95% CI, 0.65- 0.94), 0.59 (95% CI, 0.40- 0.86), 0.84 (95% CI, 0.75- 0.94) and 1.28 (95% CI, 1.06 1.55).
Xu, J., et al. 2022	*n* = 23,986 individuals from the Genetics of Iron Consortium.<br><br>*n* = 417,596 participants from the U.K Biobank and Arthritis Research UK Osteoarthritis Genetics Consortium	*HFE* (rs1800562 and rs198851)	Osteoarthritis, hip osteoarthritis and knee osteoarthritis	Genetically determined transferrin saturation was associated with knee osteoarthritis (OR:1.06; 95% CI,1.015-1.119) and hip osteoarthritis (OR: 1.56; 95% CI, 1.086, 1.229).
Qu, Z et., al. 2021	*n* = 361,141 individuals from the U.K Biobank<br><br>*n* = 455,221 participants from the U.K Biobank and Arthritis Research UK Osteoarthritis Genetics Consortium	*HFE* (rs1800562 and rs198851)<br>*TMPRSS6* (rs855791)<br>*TF* (rs8177240)	Osteoarthritis, hip osteoarthritis and knee	Genetically determined high iron status was associated with osteoarthritis in women (OR: 1.006; 95% CI: 1.00, 1.012) but not in men (OR: 1.004; 95% CI: 0.99, 1.01).
Tang, Y., et al. 2022	*n* = 48,978 individuals from the Genetics of Iron Consortium.<br><br>*n* = 336,650 individuals from the U.K Biobank	*HFE* (rs1800562 and rs198851)<br>*TMPRSS6* (rs855791)	Back pain	Back pain was associated with genetically instrumented serum iron (OR: 1.01; 95% CI, 1.00–1.02), ferritin (OR: 1.02; 95% CI, 1.00–1.04), and transferrin saturation (OR: 1.01; 95% CI, 1.00–1.01). Transferrin was not associated with the risk of back pain (OR: 0.99; 95% CI, 0.98–1.00).
Wang, K., et al. 2022	*n* = 361,194 individuals from the U.K Biobank<br><br>*n* = 48,978 individuals from the	*HFE* (rs1800562 and rs198851)<br>*TMPRSS6* (rs855791)	Liver disease	Higher iron status, by serum iron, was positively associated with risk of non-alcoholic fatty liver disease (OR: 1.89; 95% CI, 1.05–3.39) and alcoholic liver disease (OR: 1.76; 95% CI ,1.01–3.06).

	Genetics of Iron Consortium.			Serum transferrin was negatively related to liver fibrosis/cirrhosis (OR: 0.50; 95% CI, 0.25–0.97) and malignant neoplasm (OR: 0.42; 95% CI ,0.21–0.86).
Tian, T., et al. 2021	n = 48,978 individuals from the Genetics of Iron Consortium.<br><br>n = 176, 899 individuals from FinnGen	*HFE* (rs1800562 and rs198851) *TMPRSS6* (rs855791)	Primary liver cancer	There was no causal link between serum iron (OR: 1.22; 95% CI, 0.91-1.64), ferritin (OR: 1.00; 95% CI, 0.63, 1.57), transferrin (OR: 0.94; 95% CI, 0.85-1.03) and transferrin saturation (OR: 1.14; 95% CI, 0.77-1.67) and primary liver cancer risk.
Yuan, S., et al. 2020	n = 48,978 individuals from the Genetics of Iron Consortium.<br><br>n = 288,951 individuals from the Breast Cancer Association Consortium<br><br>n = 367,643 U.K. Biobank.	*HFE* (rs1800562 and rs198851) *TMPRSS6* (rs855791)	Overall cancer and 22-site specific cancers	Genetically determined iron status was positively associated with liver cancer, inversely associated with brain cancer but not associated with overall cancer or the other 20 studied cancer sites at $p < 0.05$.<br><br>The odds ratios and (95% CI) of liver cancer were 2.45 (0.81- 7.45), 2.11 (1.16-3.83), 10.89 (2.44-48.59) and 0.30 (0.17-0.53) for one standard deviation increment of serum iron, transferrin saturation, ferritin and transferrin levels, respectively.<br>The corresponding ORs (95% CIs) for brain cancer were 0.69 (0.48-1.00), 0.75 (0.59- 0.97), 0.41 (0.20-0.88) and 1.49 (1.04-2.14).
Qin, H., et al. 2022	n = 48,978 individuals from the Genetics of Iron Consortium.<br><br>n = 11,348 cases<br>n = 15,861 controls from the International Lung Cancer Consortium.	*HFE* (rs1800562 and rs198851) *TMPRSS6* (rs855791)	Lung cancer	Genetically determined iron status was not associated with a risk of lung cancer. Odds ratios and (95% CIs) were:<br>1.11 (0.92-1.34), 0.76 (0.52- 1.12), 1.09 (0.86- 1.38), and 0.91 (0.81-1.02) per 1 standard deviation increment of serum iron, ferritin, transferrin saturation, and transferrin levels, respectively.
Hou, C., et al. 2021	n = 48,978 individuals from the	*HFE* (rs1800562 and rs198851)	Breast cancer	Genetically determined iron status was not associated with the risk of breast cancer or any of its

	Genetics of Iron Consortium.<br><br>*n* = 122,977 individuals from the OncoArray network.	*TMPRSS6* (rs855791)		subtypes (OR: 0.986; 95% CI, 0.936-1.040).
Guo, Y., et al. 2020	*n* = 25,509 cases and *n* = 40,941 controls from the Ovarian Cancer Association Consortium	*HFE* (rs1800562 and rs198851) *TMPRSS6* (rs855791)	Epithelial ovarian cancer	Genetically determined serum iron level was not associated with invasive epithelial ovarian cancer (OR: 0.99; 95% CI, 0.90-1.09) or low malignant potential epithelial ovarian tumours (OR: 1.05; 95%, 0.87-1.30).
Kazmi, N., et al. 2020	Overall prostate cancer:<br>*n* = 79,148 cases<br>*n* = 61,106 controls<br><br>Aggressive prostate cancer:<br>*n* = 15,167 cases<br>*n* = 58,308 controls	*HFE* (rs1800562 and rs198851) *TMPRSS6* (rs855791) *TFR2* (rs7385804) *TF* (rs855791)	Prostate cancer	Genetically determined serum was associated with decreased risk of prostate cancer (OR: 0.92; 95% CI, 0.86–0.98)
Del Greco M, F., et al. 2017	*n* = 48,978 individuals from the Genetics of Iron Consortium<br><br>*n* = 74,354 individuals from the CKDGen consortium	*HFE* (rs1800562 and rs198851) *TMPRSS6* (rs855791) *TF* (rs8177240) *TFR2* (rs7385804)	Estimated glomerular filtration rate (eGFR)	1.3% increase in eGFR per standard deviation increase in iron (95% CI: 0.4–2.1).
Sha, T., et al. 2022	*n* = 378,635 participants from U.K Biobank.	*HFE* (rs1800562 and rs198851) *TMPRSS6* (rs855791)	Sarcopenia	Genetically-determined serum iron level was associated with an increased risk of sarcopenia (OR: 1.53, 95%; CI, 1.31–1.78).
